# Unraveling Membrane Perturbations Caused by the Bacterial Riboregulator Hfq

**DOI:** 10.3390/ijms23158739

**Published:** 2022-08-05

**Authors:** Florian Turbant, Jehan Waeytens, Camille Campidelli, Marianne Bombled, Denis Martinez, Axelle Grélard, Birgit Habenstein, Vincent Raussens, Marisela Velez, Frank Wien, Véronique Arluison

**Affiliations:** 1 Laboratoire Léon Brillouin LLB, CEA, CNRS UMR12, Université Paris Saclay, CEA Saclay, 91191 Gif-sur-Yvette, France; 2 Department of Molecular Biology, University of Gdansk, Wita Stwosza 59, 80-308 Gdansk, Poland; 3 Structure et Fonction des Membranes Biologiques, Université Libre de Bruxelles, 1050 Bruxelles, Belgium; 4 Institut de Chimie Physique, CNRS UMR8000, Université Paris-Sud, Université Paris-Saclay, 91400 Orsay, France; 5 Institute of Chemistry & Biology of Membranes & Nanoobjects (UMR5248 CBMN), University of Bordeaux, CNRS, Bordeaux INP, 33600 Pessac, France; 6 Instituto de Catálisis y Petroleoquímica, CSIC, c/Marie Curie, 2, Cantoblanco, E-28049 Madrid, Spain; 7 Synchrotron SOLEIL, L’Orme des Merisiers, Saint Aubin BP48, 91192 Gif-sur-Yvette, France; 8 UFR SDV, Université Paris Cité, 75006 Paris, France

**Keywords:** small non-coding RNA, regulatory RNA, Hfq, bacterial amyloid, protein self-assembly, membrane insertion, lipid–protein interaction

## Abstract

Hfq is a pleiotropic regulator that mediates several aspects of bacterial RNA metabolism. The protein notably regulates translation efficiency and RNA decay in Gram-negative bacteria, usually via its interaction with small regulatory RNAs. Previously, we showed that the Hfq C-terminal region forms an amyloid-like structure and that these fibrils interact with membranes. The immediate consequence of this interaction is a disruption of the membrane, but the effect on Hfq structure was unknown. To investigate details of the mechanism of interaction, the present work uses different in vitro biophysical approaches. We show that the Hfq C-terminal region influences membrane integrity and, conversely, that the membrane specifically affects the amyloid assembly. The reported effect of this bacterial master regulator on membrane integrity is discussed in light of the possible consequence on small regulatory RNA-based regulation.

## 1. Introduction

Hfq is an abundant bacterial protein that plays a central role in RNA-related regulations [1,2]. In particular, in Gram-negative bacteria, it facilitates the pairing of regulatory small non-coding RNA (sRNA) with target messenger RNA (mRNA), usually around the mRNA Ribosome Binding Site (RBS) and/or the AUG translation initiation codon [3,4,5]. This allows regulation at the post-transcriptional level, usually in a negative way [6]. Post-transcriptional modulation of gene expression can affect bacterial adaptation to changing environments, which can be of primary importance for the control of cell division, quorum sensing, or virulence of pathogenic species [7,8,9]. Indeed, almost half of Hfq-binding sRNAs regulate the expression of membrane proteins to respond to envelope stresses [10,11].

Structurally, *Escherichia coli* Hfq is a 102 amino acid (11.2 kDa) protein, which forms homohexamers [12]. The N-terminal region (NTR, 2/3 of the 102 aa) consists of a five-stranded antiparallel β-sheet capped by an N-terminal α-helix (referred to as an Sm fold, PROSITE entry PS51536) [13,14]. The β sheets, from six interacting monomers, assemble in a toroidal structure with two well-differentiated faces, a distal face and a proximal face (on which the α-helix is exposed) [1]. Both the distal and proximal faces of the protein are involved in binding nucleic acids with different specificities [12,15,16,17,18]. In addition to the well-characterized NTR Sm-like domain, the protein also has a C-terminal region (CTR), which comprises about 40 amino acid residues located at the periphery of the Sm torus on the proximal side [19,20]. Although the Hfq atomic 3D structures from various bacteria have been solved, they all lack the C-terminal region [12,15,18,21,22]. The function of this CTR region, although enigmatic for quite some time, has recently been revealed. This region functions in DNA binding and compaction, in sRNA-related roles, or in increased protein stability [19,23,24,25,26,27,28,29,30]. Furthermore, Hfq-CTR can adopt an intrinsically disordered structure and fold into an amyloid structure under certain conditions [31,32]. DNA (and to a lesser extent RNA), for instance, accelerates CTR amyloid self-assembly [25]. This CTR allows Hfq to self-assemble in vitro and in vivo [33,34,35,36], a feature common to Hfq and Sm proteins [33,37].

The Hfq-CTR is also essential in the association of Hfq with the bacterial membrane and promotion of the formation of Hfq clusters close to the cellular membrane [32,38,39]. Among different RNA metabolism components [40], Hfq assembles into helical supramolecular cellular structures to mediate functional compartmentalization close to the inner membrane of the Gram-negative bacterial cell [36,39]. This property is abolished when Hfq-CTR is absent [32]. This cellular compartmentalization could play important roles in the processes of RNA degradation and maturation, for instance by avoiding premature degradation of RNA immediately after transcription; although, this remains to be proven [36,41,42].

In the present work, we extend our previous analysis demonstrating that the CTR region of the bacterial riboregulator Hfq associates with membranes [38]. Understanding the details of Hfq–membrane interaction is important because it may play a crucial role in RNA metabolism [43,44,45]. We now report results from different structural techniques, addressing details of how amyloid fibers formed by the C-terminal 38 aa of Hfq interact with membranes of different compositions in vitro. Molecular Microscopy, including Atomic Force Microscopy (AFM) and Transmission Electron Microscopy (TEM), allow observation of the evolution over time of amyloid fiber integration into the membrane. Spectroscopic techniques, including Fourier-Transform InfraRed spectroscopy (FTIR) and Synchrotron Radiation Circular Dichroism (SRCD), reveal changes in the peptide secondary structure upon membrane binding. Solid State NMR (ssNMR) detects changes in the order and mobility of lipid chains within the membrane upon Hfq-CTR binding. Together, results indicate that the interaction between the negatively charged lipids and the amyloid fibers disturbs their stability reciprocally, which strongly affects the structure and stability of the membrane. This mutual interaction could be relevant for understanding the role of Hfq in bacterial RNA exchange. Indeed, one way bacteria use to communicate with each other is to secrete extracellular vesicles into their surrounding environment [46]. In the case of Gram-negative bacteria, these vesicles are named outer membrane vesicles (OMVs). sRNAs have been found within OMVs [38,47]. Our work suggests that Hfq could play a role in the export of sRNA, which may be considered as communication molecules, in OMV [48].

## 2. Results

### 2.1. Hfq-CTR Filaments Disrupt Membranes while, Conversely, Membranes Disrupt Hfq-CTR Filaments

**Figure 1:**
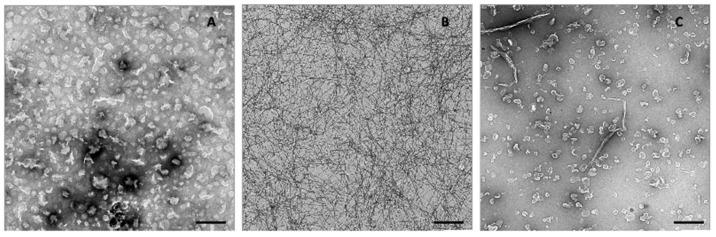
Mutual effect of Hfq-CTR amyloid fibers on lipid bilayers and conversely observed by TEM (negative staining). Transmission electron microscopy images of Hfq-CTR are taken at 2 mg/mL, alone or with EPE liposomes at 1 mg/mL. Panel (**A**): EPE Liposome alone. Note that vesicles are distorted and flattened by negative staining and subsequent TEM analysis. Panel (**B**): Hfq-CTR fibrils alone. Panel (**C**): Hfq-CTR fibrils in the presence of EPE liposomes after 5 min of incubation. Scale bar 1 µm.

Our previous analyses showed that full-length Hfq and Hfq-CTR fibrils interact with lipid bilayers producing holes in membranes, with concomitant deformation of liposomes and the generation of a multitude of small vesicles in the proximity of the fibrils [38]. This suggests protein-induced breakage of the liposome membrane. Nevertheless, the effect of the membrane on Hfq-CTR fibers was unknown. Here, we observed how lipids affect the structure of preformed Hfq-CTR fibers and vice versa, and how the lipid membranes are affected by Hfq-CTR fibrils. Using Transmission Electron Microscopy (TEM) and negative staining (Figure 1) we observed both effects, that Hfq-CTR fibrils disrupt liposomes, as expected, but conversely, that lipids induce Hfq-CTR fibril disassembly. Figure 1A shows *E. coli* polar extract (EPE) liposomes alone, and Figure 1B shows Hfq-CTR fibrils at high concentration. Figure 1C shows a solution of liposomes and fibrils after 5 min of incubation (at the same concentrations used in Figure 1A,B). This proves that there are significantly fewer liposomes and Hfq-CTR fibers than in Figure 1A,B. Because TEM is a non-quantitative technique (the spreading of the solution on grids might not be uniform), the same experiment was repeated several times to confirm the result. Moreover, we also observed the formation of thicker fibrillar structures, which is probably due to the association of fibers remaining with lipids (Figure 1C).

**Figure 2:**
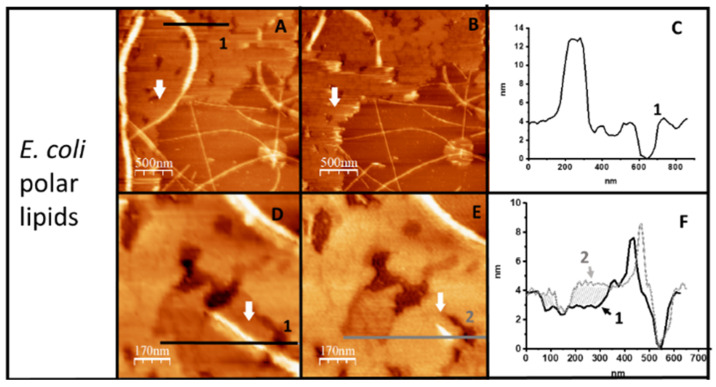
Lipid membrane and filament remodeling. Image in panel (**B**) was taken 20 min after (**A**). Panel (**C**) shows the height profile below the numbered line in (**A**). Panels (**D**,**E**) are a zoom of the same region showing the detail of filament and membrane evolution in twenty minutes (white arrows, indicate the same region). (**F**) shows the superimposed profiles in (**D**,**E**) illustrating that filament disappearance is associated with an increase in the thickness of the membrane (shaded area). Note that, as shown in Supplementary Figure S2, the monomeric peptide itself also interacts with the negatively charged lipids from *E. coli*.

The mutual interaction between the Hfq-CTR fibers and lipid bilayers was also observed by imaging a supported lipid surface immersed in solution at high resolution with AFM. Hfq-CTR fibers were deposited on previously formed supported bilayers with *E. coli* (EPE) lipids. Filaments adsorbed on the lipid surface dissolved and induced a rearrangement of the lipids. Figure 2 shows a region of the lipid bilayer that was exposed for 30 min to a solution containing fibers that were replaced by buffer before imaging. Fibers adsorbed on the surface induced the formation of holes in the lipid bilayer and the presence of the bilayer destabilized the filaments. It is noticeable that filaments lying directly on top of the mica (lower right-hand corner of Figure 2A,B) remain significantly more stable than the ones directly exposed to the lipid bilayer. This mutual effect can also be clearly seen in the Movie S1 shown in Supplementary Material: CTR filaments dissolve and simultaneously holes appear in the supported membrane. Note that if the filaments dissolve on the lipid bilayer, they remain more stable in the regions devoid of a membrane (Supplementary Figure S1); furthermore, the holes in the membrane increase in size as the filaments disappear (Supplementary Movie S2).

A closer look at the bilayer shows that the disruption of the filaments alters the surface topography. Figure 2A,B shows images of the same region taken sequentially 20 min apart. Figure 2A–C shows an area where the appearance of holes on the membrane and filament disappearance can be observed. The profile shown in Figure 2C confirms that the depth of the holes is 4 nm, compatible with holes spanning the membrane thickness. When a smaller region of the lipid surface is imaged in detail (Figure 2D,E) it becomes obvious that the membrane has undergone further reorganization and that, besides some holes, depressions 1 nm below the membrane level are present. These depressions increase their height in the vicinity of dissolved filaments (panel F), suggesting that peptides coming from the filaments insert in the membrane and raise the surface by 1 nm.

### 2.2. Membranes Break Hfq-CTR Amyloid-like Structure

**Figure 3:**
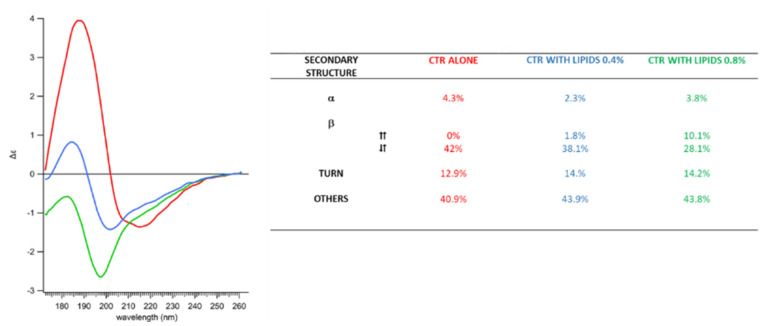
SRCD spectra of Hfq-CTR fibrils with EPE lipids. Hfq-CTR with liposome buffer (red); Hfq-CTR with 0.4 mM EPE (blue); Hfq-CTR with 0.8 mM EPE (green). Corresponding secondary structures are indicated in the table. The results obtained are in agreement with our previous reports [34,35], taking into account the SRCD spectra taken here were obtained for pre-polymerized Hfq-CTR. SRCD spectra show that the CTR is no longer amyloid-like after interaction with the membrane. Thus, the disruption of fibrils does not result in the formation of protofibrils, or oligomers that would still have an amyloid signature at 220 nm.

The precise effect on Hfq-CTR amyloid-like secondary structure was investigated using SRCD. The starting SRCD spectra were obtained with a pre-polymerized CTR peptide; then, this pre-polymerized peptide was incubated with EPE liposomes or in a corresponding SUV buffer without lipids (SUV buffer: 10 mM Phosphate buffer pH 7.5, 100 mM NaCl). Note that the use of larger liposomes could result in light scattering and for this reason, we used liposomes of 100 nm. For polymerized Hfq-CTR, the amyloid signature is found at ~220 nm (Figure 3), as expected [25,49,50]. Then we added liposomes (EPE) and observed that the amyloid signature disappeared. Specifically, we added lipids at two ratios, 0.4 and 0.8 mM for 4 mM of peptides, and observed an intermediate effect for the 1/10 ratio, and a strong effect for the 1/5 ratio. The most important difference between the spectra are amplitudes and peak shifts related to the nature of the β-sheets (parallel and anti-parallel). All three spectra indicate quite highly disordered structures (above 40%), with a fraction of α-helices (2–4%) and a significant part of β-sheets (about 40%). Increasing the lipid concentration induced a change in β-sheet composition, with a conversion from anti-parallel to parallel β-sheets.

This result correlates with the TEM and AFM experiments, for which we observed EPE lipids disrupting Hfq-CTR amyloid-like fibers. Confirmation about the possible insertion of Hfq-CTR into membranes and subsequently their orientation was then obtained with polarized FTIR and Orientated Circular Dichroism (OCD) spectroscopies.

### 2.3. Membrane-Induced Fibril-Disruption Results in the Insertion of β-Sheets

**Figure 4:**
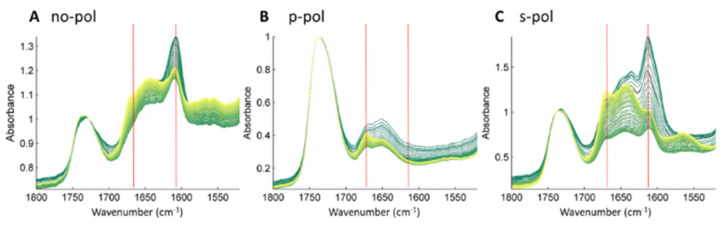
ATR-FTIR kinetics of the interaction between CTR amyloid fibrils and EPE lipids. (**A**): spectra without polarization; (**B**): with p-polarization, and (**C**): with s-polarization; The evolution of the spectra with the time is from dark-green to yellow, spectra are taken every 10 min for 15 h. The absorbance was normalized to 1 at 1730 cm^−1^. The band at 1610 cm^−1^, characteristic of amyloid fibrils, decreases during the interaction of the peptide with EPE lipids. The band at 1672 cm^−1^ that increases during incubation is probably from residual TFA used for Hfq-CTR synthesis.

To demonstrate the insertion of β-sheets into membranes, lipids were first deposited on the ATR (Attenuated Total Reflection) crystal and dried to evaporate chloroform. Water absorbs in the same area as amide I (around 1650 cm^−1^); therefore, the experiment was performed in a deuterated buffer. On top of the lipid, D_2_O buffer and protein solutions were added, and the kinetics were followed by taking IR spectra every 10 min. With ATR-FTIR, the penetration depth is around 1 µm; therefore, only molecules close to the Internal Reflection Element (IRE, here a diamond crystal) are detected. Figure 4 shows the ester band of the lipids, observed around 1740 cm^−1^ (C=O stretching vibration), and the amide I’ band of Hfq-CTR from 1700 to 1600 cm^−1^. Amide I’/II’ refers to amide I/II bands in D_2_O. The amide II’ corresponding to N-D bending band is not shown as it is centered around 1450 cm^−1^. Each secondary structure absorbs in a different area in the amide I’ band: α-helix absorbs around 1654 cm^−1^, β-sheet around 1630 cm^−1^, and turns around 1672 cm^−1^ [51]. The β-sheet structures involved in an amyloid structure usually absorb in the IR region between 1630 and 1610 cm^−1^, depending on the strength and number of hydrogen bonds, but also the number and length of β-strands [52]. In Hfq fibrils, the position of the amyloid β-sheet absorption is around 1610 cm^−1^ [35,53], indicating a strong interaction between β-sheets within the amyloid structure [52]. The evolution of the secondary structure of the Hfq-CTR fibrils can be followed by monitoring the amide I’ band signal. Figure 4A shows the kinetics of interaction between EPE lipids and Hfq-CTR fibrils. The spectra were normalized at 1730 cm^−1^ on the lipid band absorption. At the beginning of the kinetic analysis, two major bands are observed in the Amide I’ region. The band at 1660 cm^−1^ corresponds to random and/or α-helix structures, and that at 1610 cm^−1^ corresponds to the amyloid β-sheet. These two bands increase during the first phase of the kinetics, corresponding to the sedimentation of Hfq-CTR fibrils on the lipid. After this sedimentation, a second phase of the kinetics is observed with a decrease in the 1610 cm^−1^ signal and an increase in bands at 1621 and 1650 cm^−1^ (shown on second derivative spectra in Figure S1). This second phase of the kinetics is explained by the interaction of Hfq-CTR amyloids with lipids upon which a change in β-sheet conformation occurs, with an increase in the disordered structure or non-amyloid β-sheet, as indicated by the changes in these two bands. The band at 1686 cm^−1^ decreases during the incubation of the peptide with EPE, as observed from the second derivative (Supplementary Figure S3). This is not observed in Figure 4A, where the band at 1672 cm^−1^ increases, which corresponds to tri-fluoroacetic acid (TFA) traces remaining in the peptide after synthesis. The presence of the two bands for the β-sheet at 1610 and 1686 cm^−1^ is characteristic of an antiparallel-β-sheet structure [54].

SRCD results in Figure 3 reveal an evolution from amyloid fibers with initially no parallel β-sheet content to peptides containing less antiparallel β-sheets but with more unordered structures and parallel β-sheets. The same changes in the β-sheet content can be observed by ATR-FTIR. This effect correlates with the decrease in the fibril thickness and the appearance of holes in the lipid membrane shown by the AFM experiments. The timescales observed for AFM and FTIR measurements are similar. A visible change is observed in 20 min for AFM and spectra are taken every 10 min for FTIR. During the time course of measurements, the specific signature of Hfq-CTR fibrils (thickness for AFM and band at 1610 cm^−1^ for FTIR) decreases. The time required for total degradation of the fibrils is in the order of hours.

The effect of Hfq-CTR on lipids can also be observed by analysis of the ester carbonyl band at 1730 cm^−1^. There is a small shift in the maximum position of this band from 1730 to 1740 cm^−1^. This modification of the ester band is an indication that the lipid has an effect on the Hfq fibrils. The fibrils, however, also have an effect on the lipids. This effect, already observed by AFM and TEM, is confirmed by solid-state NMR and discussed in the next section.

As amyloid fibrils are oriented structures, their IR dichroism analyses provide information on their orientation. The cross β-sheet structures are perpendicular to the fibril’s long axis and the carbonyl (C=O) of the peptide bond is in the fibril’s axis. Here, the fibrils are mainly deposited on the surface, and the amide C=O is on the ATR crystal plane. For IR dichroism measurements, the IR source is polarized and absorption will occur if the polarization is in the same direction as the bond vibration. For our amyloid fibrils, this means that the band at 1610 cm^−1^ corresponds to the stretching of the C=O band in the peptide bond, and that this band will have a higher absorbance if the light is polarized on the crystal plane (s-polarization) than in the normal axis (p-polarization). A kinetic analysis was performed for both p-polarization (Figure 4B) and s-polarization (Figure 4C). For the first spectra (dark green), the effect of the polarized light can be seen on the amyloid fibrils. In Figure 4B the absorbance at 1610 cm^−1^ is lower than that at 1650 cm^−1^, and in Figure 4C the 1610 cm^−1^ band has higher absorbance than that at 1650 cm^−1^. This confirms a favored orientation of the β-sheet. For s-polarization, a decrease in the band at 1610 cm^−1^ with the increase in the 1643 cm^−1^ band is observed. Interestingly at the end of the kinetics (yellow spectra), the intensity of a new band at 1686 cm^−1^ is higher than the 1643 cm^−1^ at p-polarization but lower at s-polarization. This suggests that the new structure adopted by Hfq-CTR within the lipid membrane is oriented along the normal axis. Therefore, initially, while the C=O of the amide and the fibril’s axis are perpendicular to the lipids and parallel to the membrane, the C=O bonds of the new structure formed after membrane-induced fibril disruption are in the same direction as the lipids and inserted perpendicular to the membrane. This orientation needs, however, to be confirmed as the band at 1686 cm^−1^ overlaps with the TFA band.

**Figure 5:**
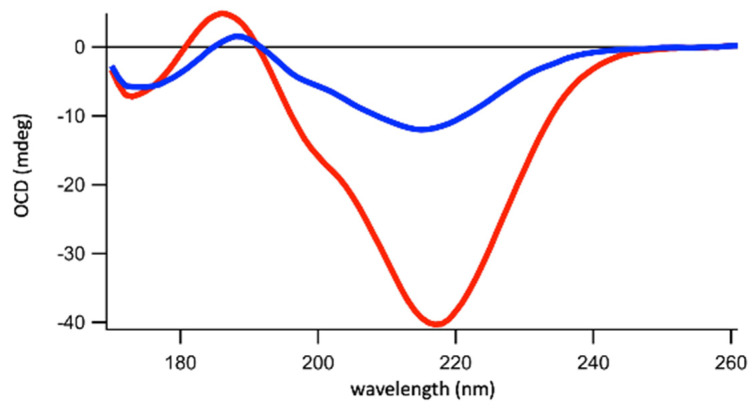
OCD analysis of the interaction between Hfq-CTR amyloid fibrils with EPE lipids. For the parallel alignment of a secondary structure with respect to the surface supported membrane or CaF_2_ surface, the CD band has a maximum negative amplitude (red curve). If the peptide structure changes its alignment and is inserted perpendicularly to the membrane plan, the value of OCD decreases (blue curve). This confirms that the peptide is inserted perpendicular to the membrane. Note that in our conditions with a bilayer, the lipids are limiting compared to peptides and probably closer or even less than the ratio 1/10 used in this figure (blue curve). This explains why the spectrum has a minimum of around 220 nm and not 200 nm.

The insertion of the peptide in the membrane was thus also analyzed using an Oriented Circular Dichroism (OCD) experiment with a lipid bilayer supported by a CaF_2_ CD cell. The OCD technique consists of overcoming the effects of alignment (as in the case of our fibers) in the CD spectra, which distorts the measurement (due to the linear dichroism signal contribution). The OCD spectra can be obtained by averaging the CD spectra obtained in different orientations of the cell. This approach has been used to analyze α-helical peptides, but it also applies to β-sheets [55]. OCD analysis results, in general, in the observation of the alignment of a peptide with respect to the membrane surface. A maximum and a minimal (negative) amplitude are observed for parallel alignment and perpendicular insertion, respectively. If the alignment of the peptide structure changes its orientation from lying on the membrane (being parallel) to insertion into the membrane (being perpendicular), the OCD signal decreases up to almost vanishing [55]. This phenomenon leads to the decrease in absorption of circular polarized light due to orientation. In other words, a reduction in interactions between the electric vector from circularly polarized light and the transition dipole of the peptide bonds (n-π* and π-π* transitions) along the β-sheets, is responsible for the variation in OCD signal amplitude. We observed that OCD amplitudes decreased when the peptide interacts with the membrane. The lipid monolayer being fully transparent to UV light, the decrease confirms that the peptide is inserted, with a high propensity of being perpendicular to the membrane (Figure 5), in agreement with orientated FTIR results.

### 2.4. Dynamics of the Lipid Membrane Is Affected by Hfq-CTR

**Figure 6:**
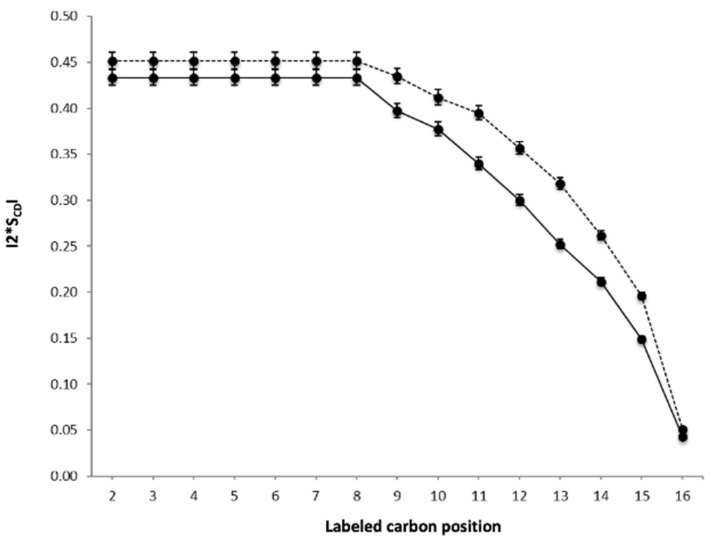
ssNMR analysis. SCD order parameters of dPOPC chains as a function of the labeled carbon position, at 310K, for a mixture of dPOPC (“reporter lipid”) and *E. coli* polar extract with and without Hfq-CTR (solid line for lipids alone and dashed line for lipids with Hfq-CTR). The presence of Hfq-CTR fibrils increases the SCD value, indicating an increase in membrane rigidity.

To further complement the biophysical assessment of Hfq-CTR interaction with membranes, we analyzed its interaction with the lipid bilayer by solid-state NMR (ssNMR). Solid-state ^2^H NMR data encode the extent to which lipid bilayers are deformed by a stress and, therefore, how lipid–peptide interactions affect membrane structure [56]. The segmental order parameters of the deuterated lipids (S_CD_) [57], which can be derived from the ^2^H quadrupolar splittings, report the relative mobility of the lipids in the presence or absence of the peptide. Figure 6 shows the segmental order parameter S_CD_ in the absence (solid line) or in the presence (dashed line) of the Hfq-CTR peptide. The segmental order parameters S_CD_ were calculated by means of ^2^H-NMR spectra, performed on lipid bilayers, made of *E. coli* polar lipid extract, and deuterated POPC (dPOPC) used as a “reporter lipid”. Figure 6 demonstrates the impact of the Hfq-CTR peptide on the lipid bilayer containing *E. coli* polar lipids, increasing the segmental order along the acyl chain of dPOPC and affecting even the inner carbon atoms of the lipid bilayer. Since the S_CD_ of dPOPC increased in the presence of Hfq-CTR peptides, the peptides have a stiffening effect on the lipid bilayer.

This result is consistent with insertion of Hfq-CTR inside the membrane bilayer, which would explain the disappearing of the fibrils observed by AFM, a result in agreement with FTIR and SRCD analyses. The stiffening effect was also observed by ATR-FTIR on EPE in D_2_O without dPOPC. The insertion in the membrane may also explain the change in the orientation of the peptide observed by ATR-FTIR, accompanied by a change in the secondary structure of the protein.

## 3. Discussion

In this manuscript, we demonstrate for the first time that the Hfq C-terminal region influences membrane integrity and, conversely, that the membrane specifically affects the amyloid assembly. We clarify the precise effect of membranes on Hfq-CTR structure and show that Hfq-CTR fibrils interact with negatively charged lipids such as those found in the *E coli* membrane (EPE). Note that, as previously shown, the minimal sequence within the CTR sufficient to interact with the membrane comprises only 11 amino acids at the very end of the Hfq sequence (SAQNTSAQQDS) [38]. This short region does not contain charged amino acids or strong hydrophobic patches, but forms amyloid fibers [38,49]. Thus, this is mainly the amyloid nature of the assembly that drives the interaction of the fibers with the membrane, even if the presence of positively charged residues within the 38 amino acids CTR may reinforce the interaction with the negative lipids [38].

In general, the interaction of amyloids with membranes promotes amyloid formation [58,59,60,61]. Here, we show that there is a complex interplay between the membrane and Hfq-CTR as the pre-fibrillized peptide is dissociated by the membrane. However, in parallel, we also observe that the monomeric peptide interacts with the membrane, which, in turn, allows the formation of patches of CTR (not fibrillar) with an amyloid-like signal (Supplementary Figure S2). A recent publication has also shown perturbations of lipid membranes when incubated with Aβ oligomeric peptides [62]. These results are comparable with ours with respect to hole formation in the membrane once incubated with peptides, but with fibrillar species in our case, and oligomeric species in the case of Aβ. Our results differ from those with Aβ and may be indicative of a functional vs. pathogenic amyloid [63]. Furthermore, the membrane here may contribute to the separation of Hfq fibrils into monomers or oligomers, to give the protein, new cellular roles.

Finally, the thickness of the fibrils deposited on the lipids slowly decreases, and this is accompanied by a decrease in the intermolecular β-sheet content (a specific signature of amyloid fibrils). In particular, we show that initially, the β-sheets of the amyloid structure are layered on the surface of the membrane, and that they then undergo a structural change and slide perpendicularly into the membrane. This explains the appearance of holes in the membrane, even if the presence of peptides in these holes has to be confirmed. Conversely, we also show that Hfq-CTR affects the membrane structure and induces higher order and rigidity in EPE lipids. This effect is observed by ssNMR for all carbons in the acyl chain, strongly indicating interactions and potential insertion of the peptide in the membrane bilayer. This result, concordant with the thinning of the infrared CH_2_ and CH_3_ stretching band, is also observed for the IR-band shift at the position of the carbonyl. These observations are indicative of the modification of the lipid chain environment. Functionally, the membrane rigidification may regulate the *E. coli* proliferation rate [64]. This rigidification could, in turn, influence the production of an extracellular polymeric substance matrix [65] or the efficiency of vesicle secretion [66].

**Figure 7:**
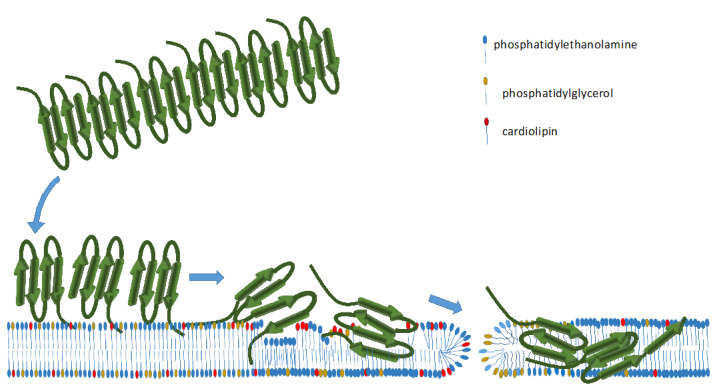
Model of the interaction of Hfq-CTR filaments with *E. coli* lipids. First, Hfq-CTR fibers interact with the membrane and then depolymerize. The first step occurs in less than 1 h, while the second step, membrane disruption and Hfq insertion, takes a few hours. The *E. coli* membrane contains both neutral and negatively charged lipids. Hole formation and peptide insertion most likely require the presence of negatively charged lipids, either phosphatidylglycerol (PG) or cardiolipin (CA). Our results suggest that negatively charged lipids destabilize the peptides in the filament and promote their perpendicular insertion into the membrane. This occurs with a simultaneous decrease in antiparallel β-sheet content. The stiffening of the membrane lipids is detected all along the carbon chains and, in parallel, an increase in the membrane thickness in the vicinity of the dissolved filaments occurs. As Hfq interacts mainly with polar lipid, Hfq-CTR insertion could induce phase separation of the PE and PG lipids. Hfq and sRNAs may also change *E. coli* membrane lipid composition [71].

The complementary information obtained from different techniques is compatible with the model presented in Figure 7 for the behavior of the filaments on *E. coli* lipids. A decrease in β-sheet content accompanied by a reorientation of the monomers with respect to the membrane was detected by SRCD and ATR-FTIR. The stiffening of the membrane lipids was detected all along the carbon chains by ssNMR and ATR-FTIR. AFM detected an increase in the membrane thickness in the vicinity of the dissolved filaments. All results suggest that negatively charged lipids destabilize the peptide assembly and promote their perpendicular insertion into the membrane. The kinetics and timescales of the modifications followed by AFM and ATR-FTIR are similar, indicating that after the first step of sedimentation of the fibrils on the surface (less than 1 h), the lipid–fibril interaction takes a few hours to reach the full depolymerization of Hfq-CTR fibrils. The difference in time scales for bacterial cell division (~20 min) and effects on the membrane (~hours) could appear surprising, but in vitro conditions are different from those observed in vivo. In particular, the conditions of crowding and confinement in vitro have not yet been taken into account [67]. Furthermore, the effect of membrane opening might be relegated to cells in a stationary phase that are not dividing, thus with no time issue. This supports our hypothesis that this mutual interaction between the lipids and the amyloid fibers could have functional relevance.

As for the functional consequence of pore formation and membrane reorganization, we suspect that Hfq may allow the export of sRNA outside of the cytoplasm by forming transient pores in the bacterial inner membrane. Taking into account the size of the pores made (few nm), an in vivo imaging method that requires high resolution is, however, required to address this question. Furthermore, the formation of holes is a dynamic process and the imaging approach will need to trap transient pores formed when they appear in vivo.

As for a putative RNA export outside the Gram-negative cell, Hfq may allow the export of sRNA in the periplasmic space and then RNAs may be exported as cargo via the formation of outer membranes vesicles (OMV) [47,68]. This should allow the exchange of genetic material in the extracellular environment between different bacteria. Preliminary results indeed suggest that Hfq controls the amount of sRNA present in OMV, but it also greatly affects sRNA stability in the cell before export, in agreement with previous reports [69]. In addition, Hfq controls the amount of OMV produced. These preliminary results must thus be confirmed and extended to several classes of sRNA (Class I/Class II) [70].

## 4. Materials and Methods

### 4.1. Expression and Purification of Hfq Proteins

Hfq-related peptide was chemically synthesized (Proteogenix, France) and prepared as described previously in Fortas et al. [32]. This peptide corresponds to the amyloid CTR domain of Hfq (residues 64 to 102) and is referred to as CTR throughout the manuscript. The sequence of the CTR peptide is: SRPVSHHSNNAGGGTSSNYHHGSSAQNTSAQQDSEETE.

### 4.2. Preparation of Small Unilamellar Vesicles (SUVs)

Lipids purchased from Avanti Polar Lipids (Alabaster, AL, USA) were dissolved at 10 mg/mL in chloroform:methanol 1:1 (v/v) on ice. The lipids used were *E. coli* Polar Extract (abbreviated EPE) and deuterated 1-palmitoyl-2-oléoyl-sn-glycero-3-phosphocholine (abbreviated dPOPC). The solvent was slowly evaporated with N_2_(g), forming a lipid film that was then dried for 30 min under N_2_ flow. Lipids were hydrated in SUV buffer (10 mM Tris pH 7.5 containing 100 mM NaCl). Vesicles formed during slow stirring at room temperature over 30 min. The solution was then passed about 30 times through a 0.2 µm polycarbonate filter (Avanti Mini Extruder) to obtain SUVs. As seen by TEM, SUV were homogeneous (averaged size was around 150 nm, see Figure 1A). The low amount of multilamellar vesicles was checked in our previous work [38]. Samples were kept at 4 °C and used for subsequent experiments within one week. For liposomes used in CD experiments, the protocol was identical, but Tris-HCl was replaced by phosphate buffer (10 mM sodium phosphate buffer pH 7.5 with 100 mM NaCl) and a 0.1 µm polycarbonate filter was used for extrusion.

### 4.3. AFM Imaging in Solution

The SUV suspension diluted at 0.4 g/L in SUV buffer supplemented with 2 mM CaCl_2_ was incubated on a freshly cleaved mica surface for one hour at 37 °C. The sample was then rinsed ten times with SUV buffer to remove excess SUVs. Supported bilayers were checked by AFM to confirm the extent of surface coverage and the quality of the lipid bilayer on the mica before protein incubation.

The proteins were added to the bilayer surface at a given concentration at room temperature for various times (from 5 min to several hours). Incubations were conducted in a sealed box to avoid evaporation. The sample was then rinsed ten times with SUV buffer to remove unbound proteins and imaged in the same buffer. The protein concentration was 0.2 g/L for the Hfq-CTR peptide.

Commercial Olympus rectangular silicon nitride cantilevers (RC800PSA), an Agilent technologies 5500 microscope (Santa Clara, CA, USA) operated in tapping mode at a resonance frequency of 15 kHz, and a 0.73 N/m spring constant cantilever were used. After the sample was installed on the AFM stage, about 30 min were required for the system to reach thermal equilibrium before commencing the AFM scans. Five to ten positions were imaged at different resolutions to ensure consistency among the observations. Images were analyzed using WSxM [72].

### 4.4. FTIR

Attenuated total reflection Fourier-transform infrared spectroscopy (ATR-FTIR) measurements were obtained with a diamond as an internal reflection element (IRE). A drop of lipid solution at 0.5 mg/mL in chloroform was first deposited on the IRE and dried with a nitrogen flux. Then, 5 µL of D_2_O from Cambridge Isotope was added on top, and finally, 0.5 µL of Hfq-CTR solution at 20 mg/mL in D_2_O was added. The system used was a Bruker Equinox55 purged with dry air and a diamond ATR device with a single reflection at an angle of 45° and closed with a golden gate chamber from Specac (Orpington, UK) to avoid evaporation and non-deuterated water vapor exchange. A spectrum, with a resolution of 4 cm^−1^, was acquired every 10 min for 15 h. For the polarization study, a gold polarizer was used to select polarization normal to the crystal plane (p-polarization or 0°) and polarization in the crystal plane (s-polarization or 90°). The data were treated with Kinetics, a custom-made program developed in SFMB laboratory (SFMB, Université libre de Bruxelles, Belgique) running under MATLAB (Mathworks, Natick, MA, USA). The water vapor signal was removed by subtracting a reference spectrum of pure water vapor with a coefficient optimized on the amide II area band (1555–1550 cm^−1^). The spectra were normalized on the ester band from the lipid at 1730 cm^−1^.

### 4.5. Transmission Electron Microscopy (TEM)

Grids were prepared as described in Fortas et al. [32] and examined at 80 kV in a JEOL JEM-1010 electron microscope and all images were recorded with a TemCam-F416 (4K × 4K) digital camera from Tietz Video and Image Processing Systems (TVIPS; Martinsried, Germany).

High magnification images were taken over large sections at random locations to avoid bias when making statistics. Liposomes were observed at a concentration of 0.1 mg/mL. Peptide fibrils were observed at 0.2 mg/mL.

The images presented were obtained with a solution of CTR peptide previously polymerized into an amyloid fiber at a concentration 100 mg/mL, subsequently diluted 1/50 the same day of the experiment to obtain a solution of CTR at 2 mg/mL. To this solution, liposomes prepared the same day were added to obtain a mixture of 2 liposomes per 1 molecule of CTR peptide, and finally these mixtures were incubated together for 5 min to 1 h (with corresponding controls).

### 4.6. Synchrotron Radiation Circular Dichroism (SRCD) and Orientated Circular Dichroism (OCD)

For SRCD analyses, measurements and data collection were carried out on the DISCO beamline at Synchrotron SOLEIL (proposal 2018027) [73]. After different incubation times, 2–4 µL of samples were loaded onto circular demountable CaF_2_ cells of 33 microns pathlength [74]. Three separated data collections with fresh sample preparations were carried out to ensure consistency and repeatability. Spectral acquisitions of 1 nm steps at 1.2 s integration time, between 320 and 180 nm were performed at 20 °C in triplicate for the samples as well as for the baselines. (+)-camphor-10-sulfonic acid (CSA) was used to calibrate amplitudes and wavelength positions of the SRCD experiment. Data analyses including averaging, baseline subtraction, smoothing, scaling, and standardization were carried out with CDtoolX [75].

For OCD, lipid bilayers were deposited on the CaF_2_ cell surface as described for the AFM experimental setup. The quality of the bilayer on CaF_2_ was confirmed previously by AFM. The peptide solution was then dispensed on top. Lipid films must be thin to minimize scattering and assure homogeneity. A CaF_2_ lid was then put on top (1 µm pathlength) preserving the humidity within the closed cell, keeping the membrane/protein sample airtight. The OCD spectra were averaged by recording CD spectra of the beam-centered cell, with the membrane/protein layer circulating clockwise every 22.5° using an automated rotating chamber [76]. This method allowed for the elimination of linear dichroism due to the alignment of the peptides in or on the membrane, resulting in an OCD spectrum as described by Chen [77].

### 4.7. Solid-State NMR Spectroscopy

2H solid-state NMR experiments were carried out on a Bruker Avance III-500 MHz WB spectrometer (Wissembourg, France) equipped with a 4 mm CP-MAS H/X probe. 2H-NMR spectra were acquired at 76.7 MHz by means of a quadrupolar echo pulse sequence, with a spectral width of 500 kHz, a π/2 pulse width of 3.9 µs, a 40 μs interpulse delay, and a recycle delay of 2 s. Typically, 2–4 k scans were recorded depending on temperature and sample. Samples were allowed to equilibrate for 30 min at 37 °C before the NMR signal was acquired at the same temperature. A Lorentzian noise filtering of 100–200 Hz was applied prior to Fourier transformation from the top of the echo signal.

### 4.8. Sample Preparation for Solid-State NMR

Appropriate amounts of lipids (with or without peptides) were dissolved in a solution of MeOH/CHCl_3_ (1:2, v:v). The solvent was evaporated under a nitrogen gas flux. The residual lipid film was dispersed in 1 mL Milli-Q filtered water and lyophilized overnight. The resulting fluffy powder was suspended into deuterium-depleted water to obtain a hydration, h, of 90% (h = mass of water over the total mass of the system (phospholipids plus water)). Samples were subjected to 3 freeze/thaw cycles (shaking using a vortex mixer, freezing in liquid nitrogen for 30 s, followed by heating at 40 °C for 10 min in a water bath). A milky dispersion was obtained and transferred to a 4 mm diameter Zirconium rotor (Cortecnet, Voisins Le Bretonneux, France).

This analysis was made for dPOPC/*E. coli* extract (30/70 molar ratio) and dPOPC/*E. coli* extract (30/70 molar ratio) co-solubilized with Hfq-CTR (1/25 peptide/lipid molar ratio).

## 5. Conclusions

In this work, we evidence that the C-terminal amyloid region of the Hfq riboregulator, which has lacked a clear function for years, allows the protein to interact with the membrane and introduce pores in the membrane. This opens a new and unexpected function for this bacterial master regulator. One way for Gram-negative bacteria to communicate is to secrete outer membrane vesicles OMV. As RNAs are found within OMV and may be considered as communication molecules [48], Hfq may thus be a major player in this process. Although the precise molecular mechanism involved in forming RNA-containing OMVs is not yet proven [68], understanding this new property of Hfq related to membrane physiology could certainly help reveal the underlying mechanism. As this is relevant to bacterial adaptation, these activities warrant further investigation.

## Data Availability

The data that support the findings of this study are available on request from the corresponding authors.

## Supplementary Materials

The following supporting information can be downloaded at: https://www.mdpi.com/article/10.3390/ijms23158739/s1.
