# Supplementary material for: Unraveling Membrane Perturbations Caused by the Bacterial Riboregulator Hfq
Source: Int J Mol Sci. 2022 Aug 5;23(15):8739. doi: 10.3390/ijms23158739 (PMC9369112; doi:10.3390/ijms23158739)
Supplement: Supplementary file 1 [file ijms-23-08739-s001.zip › supfigTurbant.pdf]

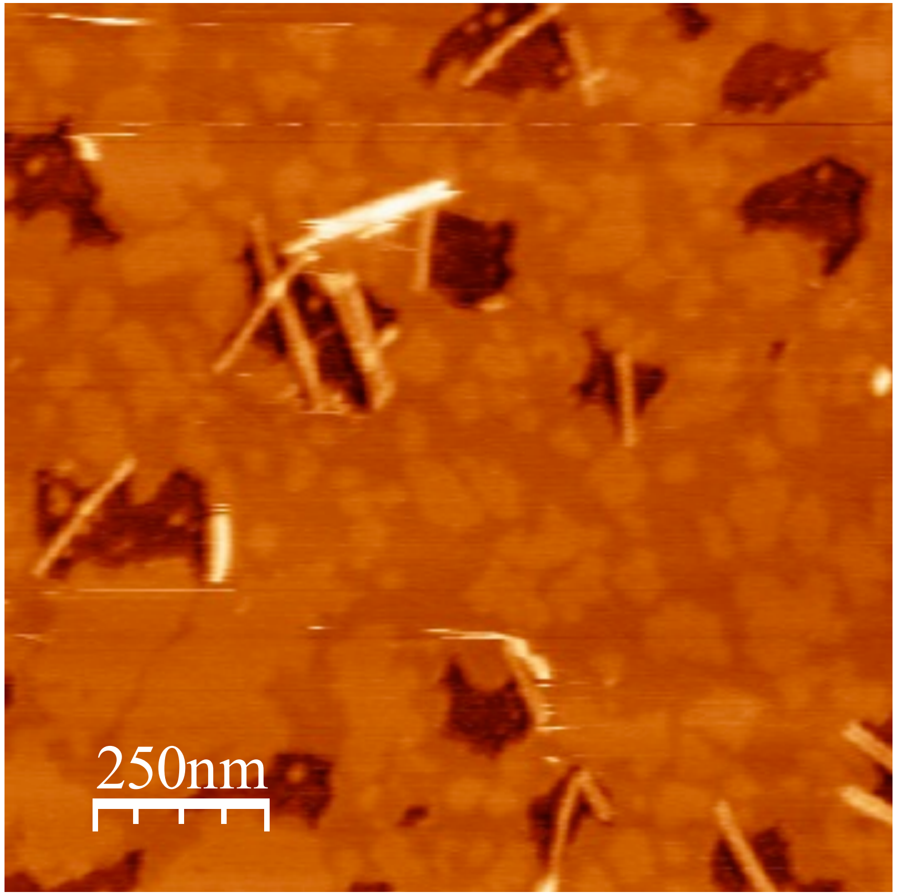

Sup Figure S1. Zoom on Hfq-CTR pieces of fibrils in the regions devoid of membranes. As shown, the CTR filaments dissolve on *E. Coli* lipid bilayer, but remain in the regions of holes.

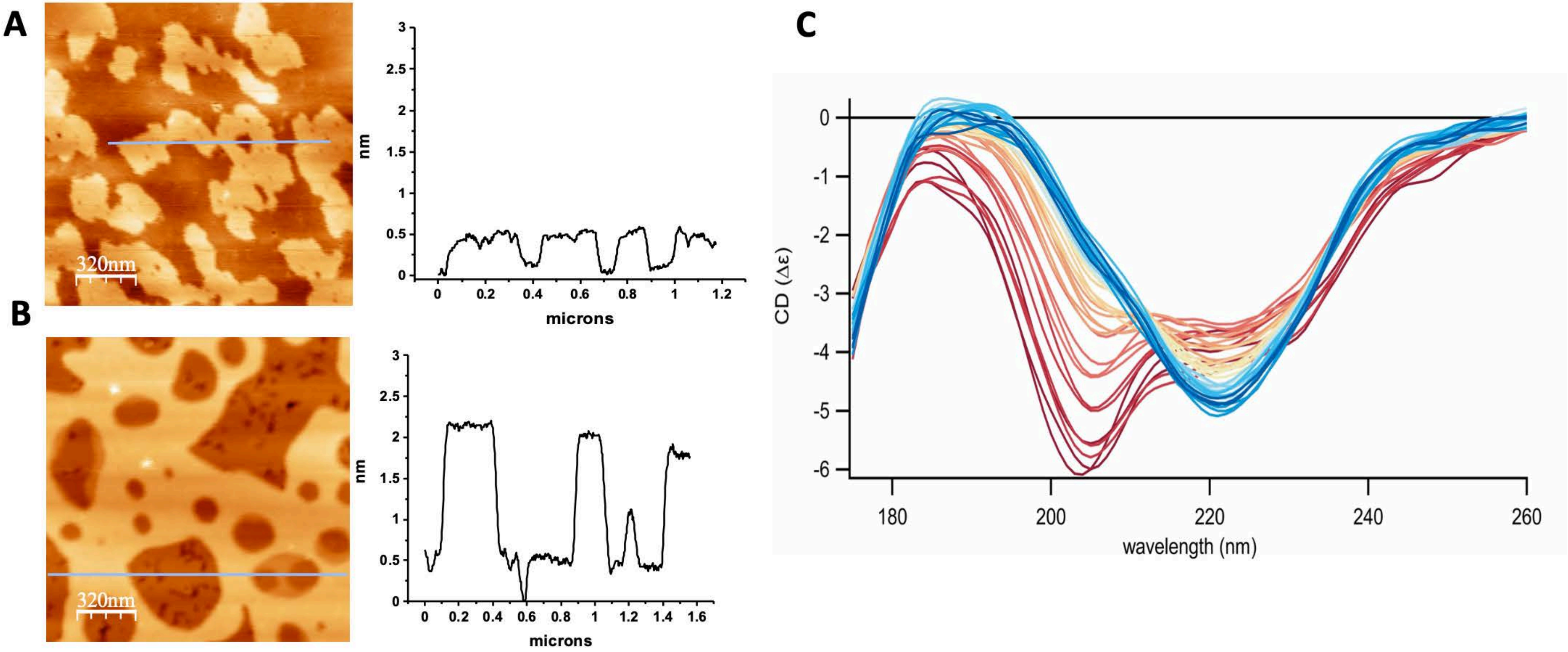

Figure S2. (A) AFM image of the surface of an *E.coli* supported lipid bilayer. The profile under the line in the image is shown on the right and indicates that the segregated lipid domains protrude 0.5 nm from the surface. (B) The same surface observed after being exposed to the CTR monomeric peptide, showing that the segregated domains reorganize and gain height, up to 2 nanometers (as shown in the profile corresponding to the line in the image). In this case, patches of CTR are formed, but no fibrils and no holes are obeserved. (C) SRCD analysis of the monomeric peptide self-assembly with EPE liposomes. SRCD reveals that the EPE lipids promotes the formation of amyloid-like patches (as seen by the transition from 205 to 220 nm).

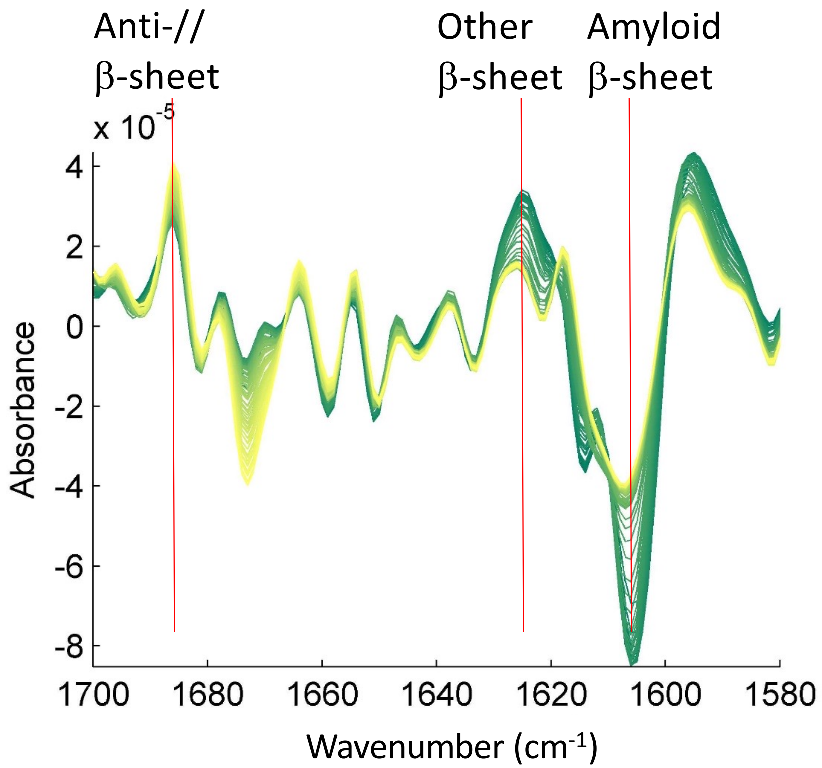

Sup Figure S3. 2nd derivative of the ATR-FTIR kinetics of the interaction between Hfq-CTR amyloid fibrils and EPE lipids. The band at 1610 cm^{-1} characteristic of β-sheet from amyloid fibrils is decreasing during the incubation of the peptide with EPE lipids, together with the at 1685 cm^{-1} indicating anti-parallel β-sheet structure and the increases during incubation of the 1625 band indicates a changes of β-sheet structure from anti-parallel amyloid fibrils to parallel β-sheet. The band at 1672 cm^{-1} is from residual TFA from Hfq synthesis.
