# Supplementary material for: Unraveling Membrane Perturbations Caused by the Bacterial Riboregulator Hfq
Source: Int J Mol Sci. 2022 Aug 5;23(15):8739. doi: 10.3390/ijms23158739 (PMC9369112; doi:10.3390/ijms23158739)
Supplement: Supplementary file 1 [file ijms-23-08739-s001.zip › supmovieTurbant.pptx]

## Slide 1
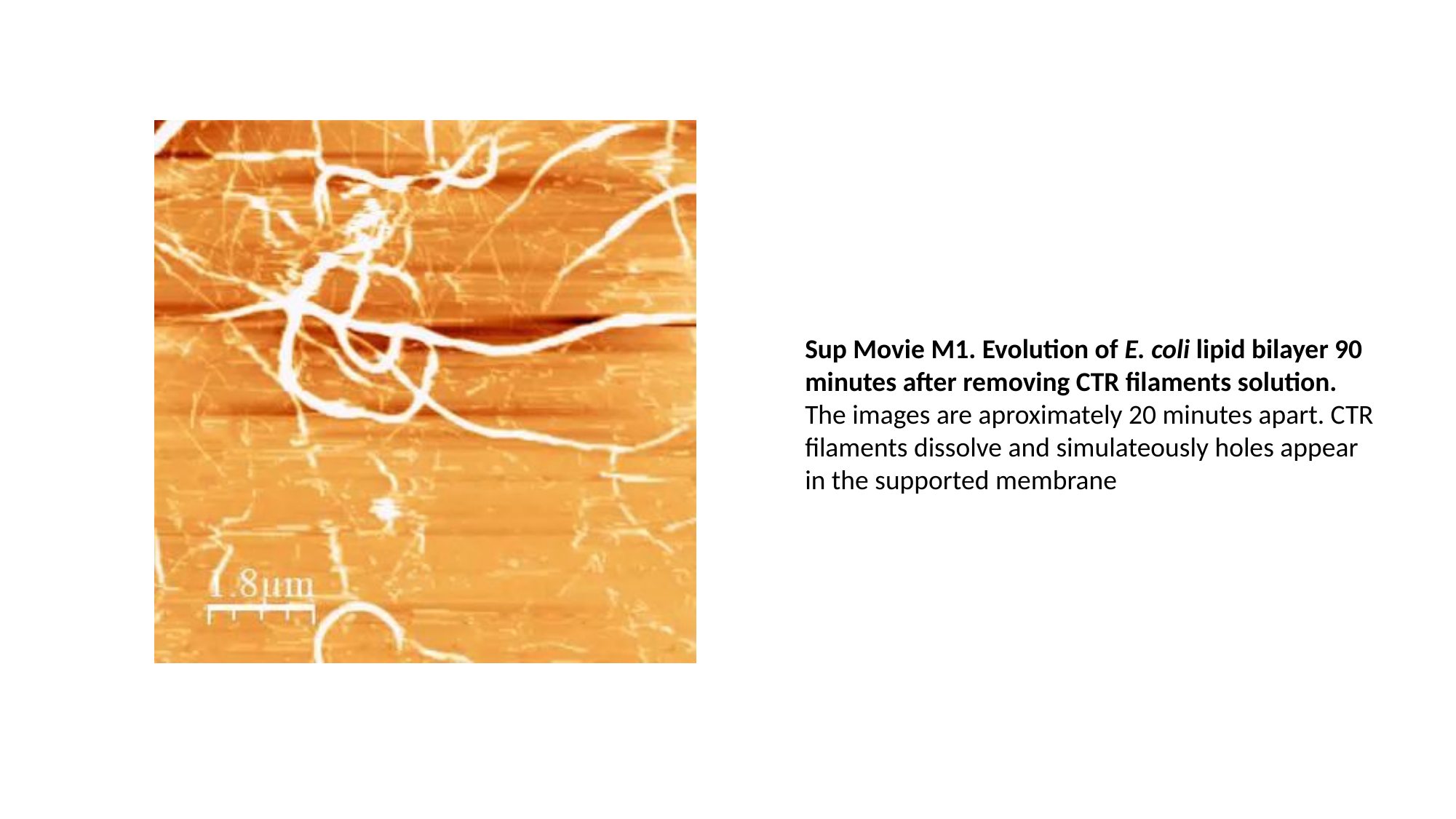

Sup Movie M1. Evolution of E. coli lipid bilayer 90 minutes after removing CTR filaments solution. The images are aproximately 20 minutes apart. CTR filaments dissolve and simulateously holes appear in the supported membrane

## Slide 2
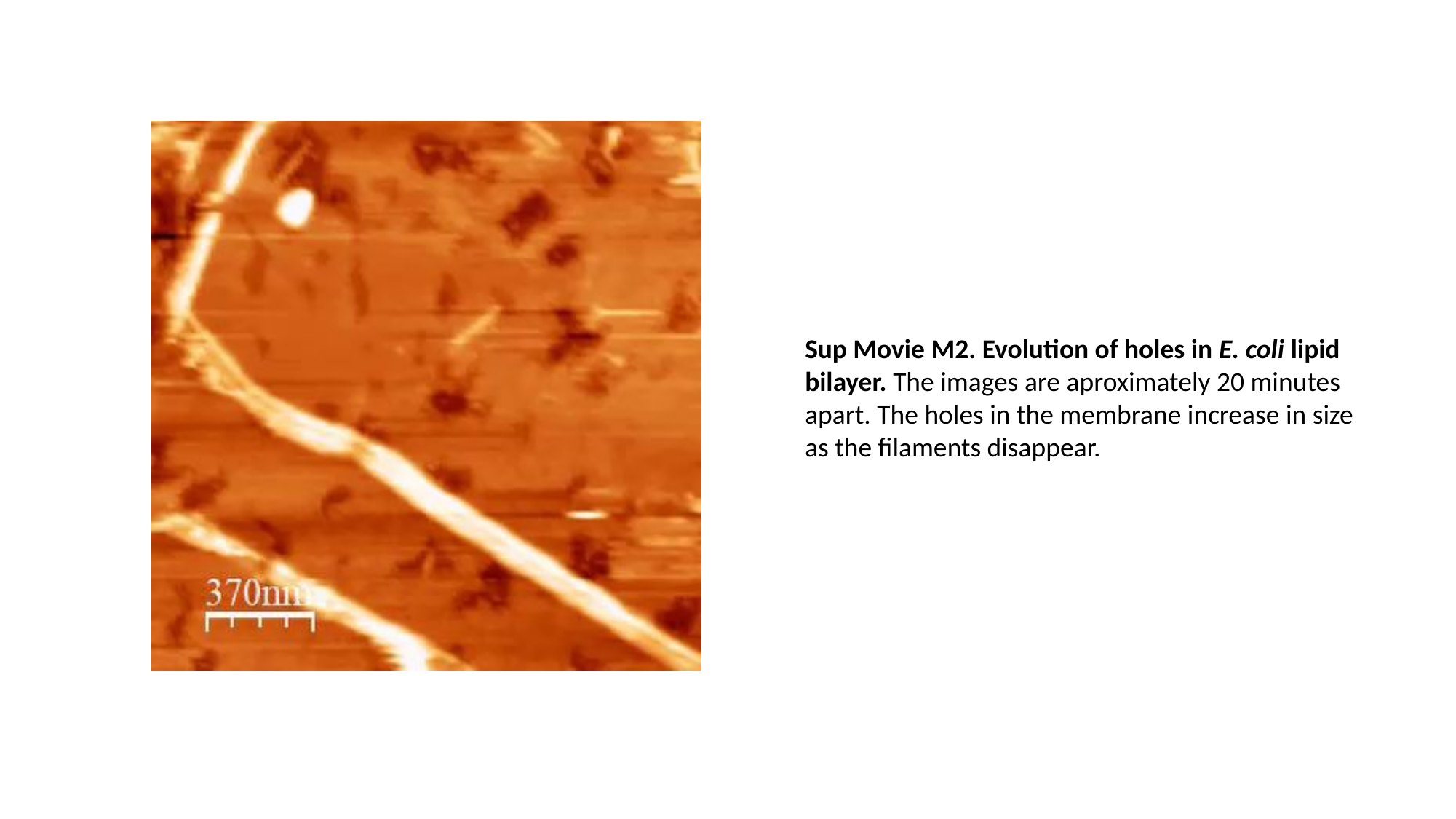

Sup Movie M2. Evolution of holes in E. coli lipid bilayer. The images are aproximately 20 minutes apart. The holes in the membrane increase in size as the filaments disappear.
